# Investigating the illicit market in veterinary medicines: An exploratory online study with pet owners in the United Kingdom

**DOI:** 10.1007/s12117-022-09463-0

**Published:** 2022-09-08

**Authors:** Monica Pons-Hernandez, Tanya Wyatt, Alexandra Hall

**Affiliations:** 1 Universitat Rovira i Virgili, Member of the Universitat Rovira i Virgili Research Group “Territory, Citizenship and Sustainability”, recognized as a consolidated research group of the Department of Research and Universities of the Generalitat de Catalunya, Tarragona, Spain; 2 Northumbria University, Newcastle upon Tyne, UK

**Keywords:** Criminogenic asymmetries, Illicit medicines, Illegal markets, Non-human animals

## Abstract

The illicit market in veterinary medicines is an overlooked issue despite threatening the health of non-human and human animals. It is thought to be increasing within the major markets of the global North due to the growth of e-commerce and social media sites. This paper examines the online market in illicit veterinary medicines through an exploratory study of the public’s online experiences as pet owners in the UK. To this end, we collected data through literature-based research and an online survey. Drawing on Passas’ criminogenic asymmetries framework, the research found that the confluence of legal, political, cultural, economic and knowledge asymmetries likely facilitate the market in illicit veterinary medicines in the UK. Our research concludes that, while previous reports suggest the illicit market is dominated by medicines to treat pets, it increasingly consists of medicines for farmed animals. This brings its own set of challenges and risks, and a pressing need for further research on the market’s dynamics.

## Introduction

Public health and the efficacy and origin of medicines are at the forefront of many people’s minds. While a number of studies in criminology have identified and analysed the illicit trade in human pharmaceutical drugs (Lavorgna 2014; Hall and Antonopoulos 2016; Koenraadt and van de Ven 2017; Baratto 2020), an overlooked public health issue is the market in illegal veterinary medicines^1^. The illicit trade in veterinary medicines constitutes a health and safety issue for both non-humans and humans with implications stretching across the globe. Not only can non-human animals become ill or die fromillegal veterinary medicines (Health for Animals, 2017), but they can also weaken food safety for non-humans and humans, increase the risk of zoonotic diseases (diseases passing from non-humans to humans like the coronavirus), and increase antibiotic resistance (which exacerbates all of the previously mentioned risks for non-humans and humans).

The estimated annual global value of the illicit veterinary market is 3% of the total veterinary market or US $1–2 billion, although this is thought to vary greatly by country (Health for Animals 2017). The main illicit market in veterinary medicines in the United States (US) and European Union (EU) is in products used to treat companion animals (pets) rather than food-producing animals (Health for Animals 2017). According to the Veterinary Medicines Directorate (VMD), this is also the case in the UK (personal communication in 2019). Yet recent seizures at the border, which increased exponentially from one in 2018 and 2019 to forty in the year ending March 2021, included a large volume of hormones, steroids, and antibiotics intended for use on both companion and food-producing animals (Vinter 2021; VMD 2021). UK officials countered that these seizures are not an indication of increased illegal activity but reflect improved cooperation between the VMD and Border Force (Vinter 2021). Regardless, the increased seizures suggest either a potential shift in the market’s dynamics from companion to farm animals or that there is substantial ongoing illegal activity about which little is known.

Although the scale of the illicit market is for the most part unknown (Vinter 2021), it is believed that the international trade in illicit veterinary medicines, especially of small packages, represents a growing problem facilitated by the growth of e-commerce. Illicit veterinary medicines are a particular problem for pet’s parasiticides (medicines used to kill parasites like fleas, ticks, lice and so forth) in the global North and their supply is increasing through unapproved online pharmacies, online marketplaces (e.g., eBay, Amazon, Alibaba), and more recently via social media (Facebook, Twitter etc.) (Health for Animals 2017). As the VMD (2014b: 1), the Executive Agency responsible for controlling the market in the UK, claims, “animal owners are now more regularly looking to buy veterinary medicines online”.

This paper explores the illegal online market in illicit veterinary medicines for pets including the public perceptions and experiences with this market in the UK with the aim to explain the market dynamics. Accordingly, drawing on data from literature-based research and an online survey with members of the UK public, we investigate the global and local scope of the issue. The UK is one of the main demand countries for illicit veterinary medicines for pets and seizures are increasing at UK borders (see Vinter 2021). Therefore, the demand side of the supply chain reflects on public knowledge of illicit veterinary medicines and people’s use of online platforms to purchase veterinary medicines in the UK. Before we detail our findings, we present the methods used to collect data and its limitations. We also introduce our theoretical framework, which draws on Passas’ criminogenic asymmetries (Passas 1999). We then detail and discuss the results. We conclude by offering some suggestions as to how the illicit market in veterinary medicines might evolve and how it might be addressed.

## Methods

Data in this paper have been collected through two research methods. The first is literature-based research, which provides the overview and wider information about the possible nature and extent of the illicit trade in veterinary medicines at the global level and in the UK. The second is a qualitative survey focused on gathering information about UK consumer trends (see Jansen 2010) in purchasing veterinary medicines online and consumer motivations for doing so. Focusing on the UK, we attempt to reflect on the major markets in the global North. Together, both methods serve to collect the necessary data on the supply and demand sides of the market. We received ethical approval from the Ethics Committee of Northumbria University in May 2020 (Submission Reference 22870).

To gather information relating to people’s purchasing behaviours and reasons for choosing to buy veterinary medicines online, the survey was created using JISC online surveys (formerly Bristol online surveys). Northumbria University?has a password protected subscription to the service. We distributed the survey online, predominantly via Twitter and Facebook from 14 to 2020 through 15 June 2020, and received a small sample of 172 responses. While it was developed and distributed with the goal of gathering information from a diverse group of pet guardians from across the UK, most of the respondents were based in England (90%). The most represented ethnic group was British (91%), most respondents identified their gender as women (78.5%), and most respondents (70%) were between the ages of 18 to 50 years old. Regarding the kind of pets reflected in the sample, the most heavily represented were dogs (67.5%) and cats (42%). Most guardians had pet insurance (61.7%). Accordingly, the findings are biased towards this sample frame.

The survey was divided into five sections consisting of open-ended, multiple-choice, and demographic questions. The first and second sections asked for the personal details of the respondents to gather better insights into their profiles and pets. The third section asked for the medical record of the pets and whether they had insurance. We also asked respondents if they knew about the possibility of buying medicines online before an open-ended question regarding which platforms they had knowledge of. Before arriving at the end of this section, we asked if they ever bought their pet’s medicines online, and, if so, they moved to the fourth section directly addressing their online buying experiences. As mentioned, in the fourth section we asked about their experience in buying veterinary medicines online, the platforms they used, and their motivations to use this service. The final section was concerned with gathering information about seller profiles. Here we collected details regarding the location of the site, if they provided an address or the VMD logo, and if they experienced any ‘red flag’ situations while online buying (see discussion below). The survey data were analysed using content analysis and descriptive statistics.

### Limitations

Before discussing our findings, we set out the limitations of this research. The exploratory nature of the research and the small sample size of the survey and its nature imply limitations in the interpretation and representativeness of the results presented below. Hence, the nature of the survey impedes distinguishing clearly those respondents referring to purchases from legal sources from those referring to illegal sources, limiting the analysis and interpretation of the data. Also, as previously stated, while the survey was designed and shared with the aim of achieving a diverse profile of participants, both in humans (e.g., location, gender, age) and non-humans (e.g., conventional and exotic pets), the profile of the respondents is largely homogeneous. The survey was shared mostly through Twitter and Facebook and the homogeneity of the sample may speak to the profile of the users that the call reached. Thus, the small sample size (n = 172) and the homogeneity of the participants impede making general claims around the extent of the use of online platforms and the motivations behind the online buying of veterinary medicines. Furthermore, the lack of literature addressing the illegal market in veterinary medicines also limits our research. To understand the supply chain of this illicit market, we had to largely rely on knowledge of the market in illicit human medicines and other illicit goods.

## Theoretical framework

To analyse the current conditions incentivising the online trade in veterinary medicines and how the illicit market might evolve, this paper uses the criminogenic asymmetries framework proposed by Passas (1999). As Passas (1999: 400) argues, “cross-border crime is a product of ‘criminogenic asymmetries’: conflicts, mismatches, and inequalities in the spheres of politics, culture, the economy and the law”. In the context of globalisation, such asymmetries are intensified, and the extent of transnational crime increases. The local and the global are more connected, but we do not have homogenised regulation, law enforcement or cultural heritages, and the global village is constrained to divergent domestic rules, traditions, and mechanisms of control (Passas 1999). As argued by Passas (1999: 402) the “asymmetries can cause crime by: (1) fuelling the demand for illegal goods and services; (2) by generating incentives for people and organizations to engage in illegal practices; and (3) by reducing the ability of authorities to control crime”.

Passas (1999) differentiates among four asymmetry realms, namely economy, law, politics, and culture. To understand the causes of this transnational crime, in this paper we also analyse knowledge asymmetries (Passas 2000). Economic asymmetries refer to the unequal economies and incomes that can lead to engaging in illegal markets either by the direct involvement in the running of the market or buying illicit goods and services (Arroyo-Quiroz and Wyatt 2019). It is in the context of economic asymmetries that knowledge asymmetries also feature because the different actors in the market (particularly the consumers) will likely have differing levels of understanding about the existence, nature, and consequences of the crime (Passas 2000). Legal asymmetries are the legislative differences between countries, which are even more relevant in a globalised world without effective international laws regulating transnational crimes. Legal asymmetries denote both weak legislation in some countries that can be exploited, and the divergent legislation between countries that can provide loopholes or sanctuaries for criminal entrepreneurs (see Pereira 2015). Similarly, political asymmetries represent weak law enforcement measures, extensive corruption levels, and limited political will and resources of the state to prevent and prosecute these crimes (Arroyo-Quiroz and Wyatt 2019). Finally, cultural asymmetries help us reflect on the differing demand for illegal goods and services in the context of global consumerism (Arroyo-Quiroz and Wyatt 2019). In other words, how and in what ways are consumers being encouraged in cultural and structural terms to engage in the market.

## The illicit trade in veterinary medicines

### Overview

The illicit trade in veterinary medicines is the illegal and unlicensed sale, distribution, and/or purchase of medicines used for animals (Smith and Whiting 2014). While it can involve legally manufactured medicines sold illegally, it can also involve medicines manufactured illegally (Smith and Whiting 2014). The World Health Organisation (WHO) (2017) differentiates between substandard, unregistered/unlicensed, and falsified medical products. Substandard medicines are those products with medical authorisation that fail to meet either national quality standards and/or specifications. Unregistered or unlicensed medical products have not gone through evaluation or approval. Falsified medicines deliberately or fraudulently misrepresent their identity, composition, or source (WHO 2017). Therefore, illicit veterinary medicines can cause harm because the contents may not be regulated, can be contaminated, or can contain none of or the wrong active pharmaceutical ingredients.

In this vein, as reported by Health for Animals (2017), the adverse effects of using an illicit veterinary medicine will vary depending on the scenario: (1) there is no active ingredient, nor harmful ingredient; (2) there is no active ingredient but one or more harmful ingredients; (3) the wrong drug is used in the illegal product; (4) the medicine contains the wrong concentration or dose of the drug or antigen. With the first and fourth scenarios, the condition of the animal may deteriorate because the medicine is ineffective, which can lead to the use of a stronger medicine (which can also contribute to development of resistance to the medicine). With the second and third scenarios, while there are a variety of harmful ingredients that have been identified in human medicines, there is lack of information regarding veterinary medicines (Health for Animals 2017). In each of these scenarios, the animal may be harmed, become (more) ill, and/or die, all of which can also increase human public health risks.

Due to the lack of literature addressing the illicit trade in veterinary medicines, we relied somewhat on literature on the trade in illicit human medicines and other illicit goods. As with other trafficked goods, illegal veterinary medicines are more likely to be produced in countries with weak legislation, where governments are hindered by corruption, and where there is the presence of organised crime groups (see Global Initiative Against Transnational Organized Crime 2021). As Shelley (2019: 224) claims, what all transnational crimes associated to global illicit trade have in common is that “[they] are conducted primarily by actors based in developing countries who cannot compete in the legitimate economies of the world, which are dominated by multinational corporations based in the most affluent countries”. For instance, as Hall et al. (2017) found that the UK market in illicit human medicines consisted of products produced largely in hubs in South Asia (India and Pakistan), East Asia (China and Hong Kong), Eastern Europe (Russia), and Latin America, with distribution hubs tending to be in the Middle East, Africa, and Central Europe (Hall and Antonopoulos 2016). Similarly, the Counterfeit Pharmaceutical Inter-Agency Working Group (2011) reported that illicit pharmaceutical drugs sold in the US tend to be manufactured in China, Indonesia, and Latin America. In a 2021 Annual Special 301 Report on Intellectual Property Protection, the Office of the United States Trade Representative (USTR) (USTR 2021) reported that India – a large hub of pharmaceutical production - as the main source of illegal medicines, followed by China, the Philippines, Indonesia, Vietnam and Pakistan. In accordance, as the VMD (2021) reports, seizures of illicit veterinary medicines in the UK in 2021 were shipped from India, Latin America, Australia, Kuwait, Singapore, Romania, and South Africa. Thus, illicit veterinary medicines seem to follow a similar pattern to that of other illicit goods including human medicines, with key production and distribution hubs based in the emerging economies of the global South (see Hall and Antonopoulos 2016).

### Online buying of veterinary medicines

“Illegal business dances to very much the same tune as legal business, using similar methods, having similar aims, and achieving similar ends” (Mackenzie 2020: 2). It is therefore unsurprising that technology has had an impact on various legal, illegal and grey markets. As Shelley (2018) claims, in the legal trade many people shop online and this is true of illegal commerce as well, which means a new illicit market is created. Taking advantage of online buying, the illegal trade expands, is anonymous, impersonal, and has limited accountability to all the actors involved in the supply chain (Shelley 2018; TRAFFIC 2015). Although the Internet and social media are now commonly used to sell illegal goods such as drugs, arms, or humans (Shelley 2018; Moyle et al. 2019), it is also used to sell grey market products, such as veterinary medicines, whose illegal status is not always apparent (Shelley 2019).

There are a number of similarities with the online illicit trade in human and veterinary medicines. Like the market in illicit medicines more generally, illicit veterinary medicines can be distributed through legitimate online pharmacies and other conventional channels but also through illegal online pharmacies, online marketplaces, and social media platforms (Hall and Antonopoulos 2016; Health for Animals 2017). The growth of e-commerce brings advantages through the availability and easy access to different goods. However, it also stimulates the illicit trade by facilitating the sale and distribution of illegal veterinary medicines (Health for Animals 2017). Online marketplaces and online pharmacies have the potential to act across a variety of jurisdictions, presenting difficulties for regulation and enforcement.

Moreover, if an operation is shut down by the authorities, it is relatively easy to set up new websites without losing a customer base (Health for Animals 2017; Shelley 2018). Indeed, many operations selling illicit medicines online run a variety of online sites simultaneously as a way of avoiding detection and increasing their market reach (Hall and Antonopoulos 2016). Health for Animals (2017) also highlight the importance of e-commerce to illicit medicine suppliers because the products are distributed through small shipments by post, meaning that it is easier to avoid detection and reduce the costs of sanctions if caught. This is a pattern identified in the illicit market for human pharmaceuticals, where traffickers lower the risks associated with large-scale seizures by spreading their products across container shipments or selling direct to consumers via small-scale postal packages (Hall and Antonopoulos 2016). Direct-to-consumer channels selling illicit veterinary medicines online are therefore likely to continue to grow (Health for Animals 2017) and evident in the recent UK border seizure mentioned earlier (Vinter 2021).

Research by Health for Animals revealed the three main factors that drive demand for illicit veterinary medicines are: (1) the features of the product, in terms of *price* and expected quality of the product; (2) the individual consumer circumstances, mostly the economic circumstances; and (3) availability and access to the product. All these factors are reinforced through online sites. Pet guardians gain easy access to cheaper products through online channels and there are low legal penalties if caught buying illicit veterinary medicines (Health for Animals 2017).

Official campaigns and international research findings suggest that a number of key characteristics can help consumers determine whether online sites are selling illicit products. In a section of the *Be A.W.A.R.E.* campaign launched by the US Food and Drug Administration (FDA), as part of the Animal Health Literacy in 2016 (see American Veterinarian 2016), the FDA (2017) asked people to watch for ‘red flags’ when buying veterinary medicines online. The FDA (2017: 1) consider the following as ‘red flags’:

- The pharmacy doesn’t require a veterinarian’s prescription for a prescription medicine.
- […] the pharmacy has no licensed pharmacist available to answer questions.
- […] the pharmacy’s website does not list its physical business address, phone number, or other contact information.
- […] the pharmacy is not based in the United States.
- […] the pharmacy’s website does not protect your personal information.
- […] the pharmacy’s prices are dramatically lower than your veterinarian’s or other online pharmacies’ prices.
- […] the pharmacy ships you medicines that you didn’t order or medicines that look very different from what your pet normally takes.

Similarly, findings from the EU Fakecare project, which investigated the online trade in illicit human medicines across the EU (including the UK), identified that illegal online pharmacies have common characteristics that can be used by consumers to identify whether they are illegitimate, including sites that do not require a prescription; present low prices and special offers for larger purchases; include text that has misspellings and grammatical errors; have no physical address; advertise POMs (prescription only medications) on the homepage; and use testimonials (Di Nicola et al. 2015). However, public complicity and the consumer demand for lower prices are also factors facilitating the illicit trade in medicines. The issue of deception is not always a clear determining factor in the demand for medicines, but sometimes a piece of a rather complex set of legal, political, economic, cultural, and knowledge asymmetries, as outlined above and explored below.

### The UK context

A legitimate veterinary medicine in the UK must comply with the following:

- In all cases, the labelling of a UK authorised veterinary medicine will be in English. However, it is permitted for companies to use multi-lingual labels as long as one of the languages is English.
- A product that is authorised for sale in the UK is one that is labelled for the UK market and bears a marketing authorisation number. A UK marketing authorisation can be identified by the letters Vm or Vh followed by a five-digit code, an oblique and a four-digit code, or by an EU prefixed authorisation number (e.g., Vm 04321/4001, Vh 05467/4007, EU/1/99/099/001–001). Only products that display an authorisation number in one of these formats should be used. However, special dispensation of unauthorised veterinary medicines may be made by the VMD [(see VMD 2014b)].
- […] The VMR [Veterinary Medicine Regulations] give powers to authorised inspectors that allow them to seize and destroy any veterinary medicine that does not comply with the regulations. Failure to comply with the VMR is also an offence and prosecutions do occur. (VMD 2014b: 1).

Veterinary medicines are distributed into four categories: (1) prescription only medicine – Veterinarian (POM-V); (2) prescription only medicine – Veterinarian, Pharmacist, Suitably Qualified Person (POM-VPS); (3) non-food animal – Veterinarian, Pharmacist, Suitably Qualified Person (NFA-VPS); and (4) authorised veterinary medicine – General Sales List (AVM-GSL).

Schedule 3 of the *The Veterinary Medicines Regulations 2013* regulates the retail supply of veterinary medicinal products. It constitutes that a POM-V may only be supplied by a veterinary surgeon or a pharmacist and must be supplied in accordance with a prescription from a veterinary surgeon (VS). The VS who prescribes the POM-V must first carry out a clinical assessment of the animal, and the animal must be under that VS’s care. A POM-VPS may only be supplied by a VS, pharmacist, or suitably qualified person in accordance with paragraph 14, and must be in accordance with a prescription from one of those authorities. An NFA-VPS may be supplied without prescription but may only be supplied by a VS, pharmacist, or suitably qualified person in accordance with paragraph 14. It constitutes no restrictions for the veterinary medicines classified as AVM-GSL but a responsible approach to the supply of these medicines is still expected (National Office of Animal Health (NOAH) 2020). Finally, the Small Animal Exemption Scheme (SAES) permits certain medicines to be placed on the market without a marketing authorisation (NOAH 2020). Thus, “[a]ll veterinary medicines administered in the UK must be granted an authorisation by the VMD. The only exceptions are veterinary medicines sold under the Exemption for small pet animals” (VMD 2014b: 2).

As mentioned above, it is believed that in the UK the buying of veterinary medicines online is increasing in popularity (VMD 2014b). While the buying of veterinary medicines over the internet is allowed in the UK, the VMD encourages the potential buyer to ensure that the site is based in the country (VMD 2014a). To aid the customer to use a safe and reputable online pharmacy, the VMD has implemented an Accredited Internet Retailer Scheme (VMD 2014a). The Accredited Internet Retailer Scheme is a voluntary scheme that a reputable online supplier of veterinary medicines can obtain for free (Veterinary Record 2012). The VMD awards a unique logo to each supplier, which contains an ID that indicates that the retailer has met the requirements of the VMD (Veterinary Record 2012). A customer can check if an online animal medicine retailer is accredited by “clicking on the logo” (Veterinary Record 2012: 551) or by checking the Government website, where the full list of accredited online retailers is available. However, it is not known if it can be or is currently being falsified, similarly to the Green Cross logo with human pharmaceuticals (see Hall and Antonopoulos 2016) or the Canadian flag in online pharmacies in the US (see FDA 2018). In the case that the internet retailer stops meeting the requirements of the VMD, the VMD will proceed to withdraw the accreditation and the provided ID logo (VMD 2012).

There is a lack of data relating to the global flow of illicit veterinary medicines in general. As mentioned, recent seizures in the UK indicate that packages of veterinary medicines headed to residential addresses were sourced from across the world, including Australia, India, South Africa, and Latin America (VMD 2021). This suggests that like the illicit market in medicines more broadly, the illicit veterinary medicine supply chain is widely dispersed in space and takes advantage of the global commerce offered by the internet (Hall and Antonopoulos 2016). Thus, building on the similarities with other illicit goods, it is likely that sales of illegal veterinary medicines through online sites and social media platforms is increasing (see Shelley 2018). In the following pages, we detail the findings of the UK public opinion survey in relation to online buying.

## UK public’s experiences of online buying

One of the most relevant findings that was gained through the survey is that—contrary to what was expected—online buying of veterinary medicines is not a common practice within our sample. Only 58% (98 out of 172) of the people had *heard* about the possibility of buying veterinary medicines online and 26% (46 out of 172) had *bought* veterinary medicines online, with half of them doing so regularly. From the participants who had not heard about the possibility of buying veterinary medicines through online channels, half of them would not engage in this practice, mostly because of a lack of trust that the medicines would be authentic indicating a suspicion of illegal activity. However, the fact that our research did not find that the online buying of illicit veterinary medicines is growing, it could be due to the small sample size and the homogeneity of the respondents.

Despite the unpopularity of buying veterinary medicines online within our sample, some valuable information can be extracted from the responses given by the participants who did buy or are buying veterinary medicines online. First, is the role played by veterinarians in leading the pet guardians to use online sites. Most of the participants who bought veterinary medicines online (26% of our total sample) were familiar with the possibility of buying veterinary medicines for their pets through online research (52%), but it is worth noting that 26% of respondents were aware of this possibility because of a recommendation by their veterinarian. As seen through the responses given, veterinarian recommendation was the second most popular source of information about the online market in veterinary medicines, confirming what has been reported by Health for Animals (2017). Contrarily, spam email was not highlighted as a source of information for pet guardians buying medicines online.

Second, in relation to the platforms used, the most predominant sites to buy were online pharmacies, accounting for 94% of the responses. Social media (Facebook) was almost unheard of and/or unused, and online marketplaces (Amazon) did not appear as regular sites of online buying of veterinary medicines (27%). Thus, while it is true that veterinary medicines can be bought through social media platforms (Facebook, Twitter, Instagram etc.) and online marketplaces (Amazon, Alibaba, eBay, and so forth), within our sample population these are not the most common places used to buy medicines for pets in the UK.

In line with the most common pets owned in the UK (see Statista 2021) and the most common pets cared for by our participants (dogs and cats, as reported above), the research found that the majority of the medicines bought online were for dogs and/or cats. This finding, along with the sample of the pets owned, does not provide enough information about the usefulness of or dependency on online buying for ‘exotic’ pets (i.e., reptiles, birds, and fish) which could be linked to these species being less common pets.

Finally, in relation to online buying, we found that the main motive for turning to online buying of veterinary medicines is the *price*. As reported by the participants of the survey, 85% who use this service do so because of the price difference between buying medicine online and buying it at a veterinary practice. Also, convenience (44%) and the availability of products (30%) were motives, with only one person referring explicitly to the COVID-19 lockdown measures in place in the UK at the time of carrying out the survey. While these results are significant, more research is needed to explore a wider sample that includes exotic pets, pets without insurance, and rural-urban differentiation, to see how these variables affect the consumer trends in purchasing veterinary medicines online.

### #VetMeds

Among the findings in relation to the veterinary medicines bought online, the survey provides information about the types of medicines bought online by the pet guardians represented. First and foremost, nearly 60% of participants who shop online use this service to buy flea/tick collars or spot on treatments (26 of the 46 participants who buy medicines online). Following these are worming treatments (22%), pain relief medications (13%), creams, liquids, or tablets for dental, respiratory or skin infections (13%), and eardrops and arthritis medication (11%).

It is worth noting that *all* the participants who shop online claimed that they had been asked for a prescription when buying POM-V and POM-VPS medicines online and that they had been asked for information about their pets when buying NFA-VPS online. However, 11% of participants did not know if the medicines that they had bought online were POMs and 33% did not know if they were NFA-VPS. This raises the possibility that some participants bought prescription medicines but had not been asked for the necessary information by the online site. Also, it is important to highlight that one respondent said that online buying was helpful because [their] “vet is reluctant to provide long standing prescriptions making regular purchases more difficult”. This exemplifies that the participant was buying online without being asked for the required vet prescription.

In terms of the appearance or packaging of the medicines bought online versus the ones bought at private veterinary practices (an indication of possible illegality), most of the participants did not notice any difference or result (65% − 29 of 46 participants). Twelve and a half percent reported differences but only related to the quantity of the medicine received (participants were able to buy more online than if they had purchased from their veterinarian). Similarly, in terms of the results expected, 85% of the pets did not experience adverse or unexpected reactions. Only 11% of participants reported cases of the medicine being ineffective or producing adverse reactions (five participants). In one case, a dog developed neurological symptoms after using a flea treatment bought online. From the cases of unexpected reactions, the medicines were bought on Amazon (60% - three participants) and the online pharmacy website, Animed (40% - two participants).

Overall, the survey found that online buying is not the norm and is more commonly used for minor treatments. Only two participants reported using online sites for ongoing treatments or for buying medicines that need an ongoing prescription.

### UK legislation compliance

According to UK legislation, buying veterinary medicines online is permitted, however, people are encouraged to buy from UK sites. As found, 80% of the UK pet guardians surveyed bought from a UK site whereas only 2% did not, and 18% did not know (36, one, and eight participants respectively). From this 80%, 97% reported that the site provided a UK address and the other 3% reported that the medicine was coming from the EU.

As explained above, the VMD created a scheme where online sellers can receive accreditation if complying with UK legislation. However, most of the participants did not know about the existence of this accreditation and the existence of the logo (85% − 39 of 46 participants). From those that were aware (15% - seven participants), almost 30% reported that whether the site had the logo or not did not have an impact on their decision about from which site to buy veterinary medicines. Moreover, 85% of participants reported that the site did not have or that they did not know whether the site had an accredited practitioner available for any queries about the medicine. However, the participants who did know about the availability of a practitioner reported being happy about the service provided.

### An illegal market of veterinary medicines

As a general finding of the survey, the most common red flags related to the illegal market of veterinary medicines (e.g., not asking for a prescription, different appearance of the product, or receiving the wrong medicine) had not been experienced by most of the participants. Only five respondents reported negative experiences with online buying. The situations experienced by these five participants were that the website looked ‘dodgy’, that they had not been asked for a prescription, or that they then received spam emails trying to sell online veterinary medicines. Overall, our survey respondents seemed aware (or suspected) that there were risks of fraudulent medications if buying them online (even if they had not been aware before of the possibility to buy medicines online).

## Discussion

### Criminogenic asymmetries

In the context of globalisation, where legal businesses exploit the new possibilities available to expand their market reach, this research attempted to highlight how illegal enterprises move in the same direction (Makenzie 2020; Passas 2003). Since our survey results offer little information on the nature and scale of illicit veterinary medicines online, we can only speculate as to the dynamics of this illicit market based upon the visible veterinary medicine market, the illicit human pharmaceutical research, and recent illicit veterinary medicine seizures.

We suggest, following the idea of Passas (1999), that the illegal market in veterinary medicines is likely to be reinforced and fulfilled by an *asymmetries chain* (see Table 1). Following the supply chain—source-transit-destination—the research finds that in every step of the trade there is an asymmetry potentially facilitating the possible movement of illicit commodities from one step to another, one reinforcing the next. In our hypotheses, we argue that the illicit online market for veterinary medicines responds to asymmetries in the realms of law, politics, economy, culture, and knowledge. We suggest legal and political asymmetries could play a role in manufacturing and shipping the illicit veterinary medicines to the destination market in the UK. Also, the destination markets are not chosen randomly; the cultural asymmetries along with the economic asymmetries are leading the illicit medicines toward those destinations where there is the potential to reach more buyers. Finally, once the veterinary medicines reach the destination market, the economic and knowledge asymmetries come into play resulting in a demand among those who want to save money on their pet’s medicines and/or are not aware of the risks online buying might have. Each of the asymmetries and their role within the chain are detailed and discussed below. Overall, the discussion offers an overview of how the asymmetries between countries may fulfil the supply and demand of illicit veterinary medicines.

**Table 1:** Asymmetries chain of the illicit trade in veterinary medicines

ASYMMETRY	STEP IN SUPPLY CHAIN		
	**Source**	**Transit**	**Destination**
**Legal**	• Weak regulation and oversight of manufacturing veterinary medicines • Weak regulation and oversight of online commerce	• Weak inspection regime of shipments • Differing licensing and authorisation of medicines • Online platforms having differing regulations and oversight	• Stronger regulation and oversight of manufacturing veterinary medicines • Stronger regulation and oversight of online commerce • Low penalties for small illegal shipments if caught
**Political**	• Lack of political will to address regulation gaps • Limited capacity to enforce existing regulation • Corruption	• Limited international cooperation	• Political will evident in legal frameworks and spot inspections
**Cultural**	• Manufacturing of medicines takes place in areas where there is not necessarily a demand for pets		• Pets are viewed as family members with the expectation they receive medical care • Online shopping and consumerism in general are normalised and easily accessible
**Economic**	• Cheaper to produce medicines • Lower incomes / higher levels of poverty		• Higher levels of disposable incomes in general • Internal economic asymmetries
**Knowledge**	• Knowledge of the illegality of the veterinary medicines and their ingredients		• Internal asymmetrical knowledge of the risks of illegal veterinary medicines by practitioners and pet guardians • Internal asymmetrical knowledge of the warning signs of illegal veterinary medicines at online sites

### Legal asymmetries

The illicit market in veterinary medicines online is seen as a low-risk and profitable activity (Health for Animals 2017). As noted above, offenders can exploit legal asymmetries at source and transit points to both manufacture the illicit medicines and ship them across borders. At source, as found through the literature review, the manufacturers use countries with weak legislation to produce the illicit medicines. Accordingly, the lack of legislation in some countries facilitates the production of veterinary medicines at unregulated sites (Health for Animals 2017). As happens with other illegal commodities, this illegal market exploits the weak legislation and price differentials in some countries to produce illicit goods (Global Initiative Against Transnational Organized Crime 2021).

At transit, on the other hand, weak legislative bodies are used to move the medicines to their destination markets. Suppliers exploit mismatches between national legislation and the weakness of international legislation to transport their products (see Passas 2000). For example, medicines can be legally produced in one jurisdiction before being shipped to another with different licensing laws. This process has also been compounded by the impact that online selling has had on the illicit market of veterinary medicines. As reported by Health for Animals (2017), social media platforms, online marketplaces and online pharmacies have the ability to operate across jurisdictions, which in turn boosts the already existent legal asymmetries. Tied to this comes the possibility of shipping small packages, which are difficult to detect and where penalties for illegal activity are very low. Thus, the internet has expanded the legal asymmetries illicit veterinary medicine trader’s exploit. Further, weak regulation of online sites is also exploited. As mentioned, if the authorities close an online site, it is easy to set it up again without losing the list of customers (Health for Animals 2017).

### Political asymmetries

As explained above, political asymmetries represent weak law enforcement measures, the presence of corruption, and the resources available within the state to prosecute crimes (Arroyo-Quiroz and Wyatt 2019). Here, the political asymmetries closely overlap with the previous legal asymmetries. As demonstrated by the activities of the UK’s VMD, there is an increasing will to govern the online trade of veterinary medicines, and the illicit market of veterinary medicines more generally, in the global North. The VMD tries to educate potential consumers of online veterinary pharmacies in identifying illicit sites and medicines. Also, it launched the accreditation scheme to help customers to detect online sites that are complying with the regulations, and through the VMR can authorise inspectors to seize and destroy veterinary medicines that do not comply with the regulations, as happened in various prosecutions carried out in the last year (see VMD 2021). Further evidence can be seen through the *Be A.W.A.R.E.* campaign launched by the FDA and, drawing similarities with the illicit trade in human medicines online, the EU Fakecare project. However, while governing the online trade in veterinary medicines is starting to move onto the agenda of some countries affected by the illicit market of veterinary medicines, this political will to govern the illicit trade is not seen throughout the world, impeding the international cooperation needed to disrupt this illegal market. As explained by Vinter (2021) and the VMD (2021), distribution hubs of illicit veterinary medicines are strategically placed within countries with weak regulatory and enforcement measures. Thus, this situation seems to indicate that some of these countries might have a weak regulatory oversight of the illicit market in veterinary medicines.

Moreover, the increase of selling illicit veterinary medicines online provides further political asymmetries in relation to the political will to govern online markets. As seen, online marketplaces and online pharmacies act across jurisdictions, making it difficult to enforce legislation, if it exists. Thus, countries need the political will to both draft and enact regulation that governs online commerce and to then enforce existing regulations. Such political will and enforcement capacity appears to vary across jurisdictions. Online pharmacies and online marketplaces evade enforcement efforts partly through the use of shipping veterinary medicines in small-packages sent to residential premises, which makes detection and interception difficult. In turn, this provides a lucrative illicit market with low penalties in the rare cases that are discovered.

### Cultural asymmetries

As mentioned above, cultural asymmetries can facilitate the demand and thus increase the flow of illicit goods (Arroyo-Quiroz and Wyatt 2019). In this research, cultural asymmetries, when combined with economic and knowledge asymmetries, set the context in which the online *selling* of illicit veterinary medicines might thrive. Developing on the work of Passas (2000), we can see a shifting cultural landscape in relation to pets, pet guardians, and pet health. This landscape has changed not only because of global mass media fostering consumerism, but also the increasing reach of social media and online cultures in normalising the search for medicines online. Here we can see some pet guardians in the UK departing from the accepted social rules (e.g., buying medicines from private vets) and moving online to buy veterinary medicines.

Furthermore, cultural asymmetries exist in the differing relations with the same animal species in different countries (see Srinivasan 2013). In the UK, dogs are seen as pets, as human companions, and therefore part of human life. This is of importance here because in the UK and other countries in the global North, dogs (along with other companion animals) have acquired an elevated cultural status that in some cases is almost comparable to human beings (Nast 2006; Power 2008). For example, in cities like New York, Paris, or London there are services and activities targeted exclusively at dogs and their human companions, like beaches for dogs, hotels, cafes and restaurants, and retail stores, or activities like *doga* (yoga with one’s dog) or *furry fandom* (ballroom dancing with dogs). It is in these contexts where companion animals have achieved a superior status (see also Cole and Stewart 2014). Companion animals are often regarded as members of the family for whom one would do anything to provide the care they need even if it cannot be achieved with the economic means available to the guardian. It is within this cultural context that is asymmetrical to other countries that the illicit online market in veterinary medicines for pets potentially begins to thrive.

These cultural conditions—of global consumerism, the promotion and legitimation of online medicine markets, and the elevated cultural status of pets in the UK—help us to understand how deviance can become thinkable in the UK veterinary medicine market for companion animals. In many global North countries, there are efforts to expose and formulate ethical responses to the subjection and exploitation that non-human animals suffer (McCance 2013). In these cultural contexts, non-human animals are social beings who should not be harmed and should be taken care of. Thus, there are major markets in the global North leading to the demand for illicit veterinary medicines for pets (see Hamilton and Taylor 2017). However, the cultural conditions do not exist in a vacuum but combine with economic and knowledge asymmetries to drive the market.

### Economic asymmetries

In the UK context of the illicit market in veterinary medicines, economic asymmetries refer to the unequal economies and incomes that can lead to buying illicit goods and services. Economic asymmetries are found to have two levels of impact at the macro and micro level. At the macro level, economic asymmetries are close to cultural asymmetries in determining the demand for illegal veterinary medicines. Here, linked to the cultural asymmetries, countries in the global North with larger companion animal markets have economic asymmetries at a macro level that shape the flow of illegal veterinary medicines. In other words, some countries have a larger proportion of individuals with disposable incomes (as explained by Nasr 2006), who have pets and take care of them.

In addition to a macro level economic asymmetry, a micro level asymmetry refers to the economic status of the individuals who engage in this market. In accordance with what has been found in previous reports, our survey found that the main motive driving the buying of veterinary medicines online in the UK is the price difference between the medicines bought online and the medicines bought at the veterinary practice, with the expectation that the medicine will achieve the same results. Thus, along with what has been reported by the Health for Animals (2017), the FDA (2017), and the VMD (2014b), this research finds that the reduced costs of online medicines are the main reason why individuals turn to online pharmacies or online marketplaces. At the micro level, and according to the findings from the survey, the low number of responses reporting online buying within our sample population might perhaps be related to the number of surveyed individuals with pet insurance covering the expenses at the veterinary practices. However, having (or not) pet insurance is also an example of micro level economic asymmetries as not every pet guardian can afford insurance.

Therefore, the cultural asymmetry accounts for the prevalence of companion animals in the global North and the expected care a pet should receive in Northern countries, and thus where illicit veterinary medicines are in demand. Meanwhile, economic asymmetries at a micro level pressure individuals with limited economic means to turn to a cheaper online purchase as they do not have the funds to cover the conventional buying of veterinary medicines, either at the veterinary practice or by having insurance for their pet. Along with this, some pet guardians might not want to pay full price for the medicines because they believe the efficacy of the medicines bought online will be the same. For some, they are unaware that there is a risk of purchasing illegal veterinary medicines when buying online. This lack of knowledge or concern about the potential negative effects of online buying is further developed in the following section.

### Knowledge asymmetries

Initially, four asymmetries were proposed, but Passas (2000) argues that in some cases new asymmetries are needed to understand the causes of crime. In this vein, Passas (2000) includes knowledge asymmetries—incomplete understanding or misinformation—as leading individuals to engage in crime. This research reveals that such knowledge asymmetries are likely impacting on the illicit market in veterinary medicines, both in relation to practitioners and pet guardians.

Practitioners, as reported through our survey, play an important role in guiding pet guardians buying behaviour towards the online purchase of veterinary medicines. While it might help the pet guardian to find an affordable option for their pets, sometimes the lack of understanding of what buying online should entail (i.e., prescription verification etc.) or the potential risks associated with online buying can inadvertently fuel the illicit market. The lack of understanding by some pet guardians can lead not only towards online buying but also towards having trouble in identifying which sites are trustworthy and which are not. But as our survey reveals, some pet guardians in the UK do not seem to have a complete understanding of the risks associated with this practice, how to identify them, and the tools accessible to them to identify suspected illicit online pharmacies and sellers. This lack of knowledge within the population can then be exploited by the illegal sellers who are looking to expand their illegal business.

## Conclusion

The market in illicit veterinary medicines is an under-researched area within criminology. While an increasing body of knowledge related to illicit human medicines has been developed, illicit veterinary medicines are largely overlooked. Despite the lack of knowledge available about the nature and extent of this market, the authorities in the UK, and other countries in the global North, started noticing an increase in seizures of these illicit goods, likely indicating an increase of online buying of veterinary medicines among pet guardians. Accordingly, through literature-based research and the responses collected through an online survey, this exploratory research focused on the online market in illicit veterinary medicines in the UK.

The data obtained were analysed using the criminogenic asymmetries’ theory. According to this theoretical framework, cross-border crimes, such as the market in illicit veterinary medicines, are the result of criminogenic asymmetries. These are the differences and inequalities between and within countries in the areas of law, economy, culture, politics and knowledge. Therefore, this research analysed the data in accordance with these five spheres. As discussed above, the asymmetries likely determine the flow of the illicit veterinary medicines and the actors at various points in the supply chain. Legal asymmetries seem to be exploited at source points to manufacture the illicit veterinary medicines in countries with weak regulations and at transit points where offenders take advantage of the legal mismatches across jurisdictions to ship the veterinary medicines. Along with this, the research found that the increase in online buying also creates the possibility of exploiting weak regulations of online sites, which again differ based upon the country where the site is based and where the site is being used. Closely related to the legal asymmetries are the political asymmetries; asymmetrical political will and capacity to govern the online market of illicit veterinary medicines means the illicit market can operate unhindered in some spaces. As the data collected highlight, some of the most affected countries have policies in place to help potential consumers to identify the illicit goods. However, this political will does not extend to other countries where weak law enforcement measures are exploited to manufacture and ship the veterinary medicines.

Moreover, cultural asymmetries are increasing the demand and flow of illicit veterinary medicines. As found, global consumerism, the promotion and legitimation of online medicine markets, and the elevated status of pets in Northern countries, helped us to understand the increase of buying veterinary medicines online in the UK. Linked to these, economic asymmetries within the UK were found to encourage online buying. Therefore, as found, macro level economic asymmetries exist where people in the richer global North choose to spend their income on companion animals. On the micro level, economic asymmetries within countries seem to foster the demand in two ways: (1) those with less income but with the same cultural need of covering the demands of pets’ health; and (2) those who have the income to pay for the medicines but choose to buy them online without knowing the risks associated with it. In this vein, we added knowledge asymmetries to emphasize the findings in relation to the potential lack of knowledge on the part of some practitioners as well as pet guardians of the risks to the health of the pets and the guardians posed by the online buying of veterinary medicines.

Overall, the research found that it is the confluence of all these asymmetries that likely facilitates and fulfils the market in illicit veterinary medicines with the Internet providing a space where the demand for these goods can reach a larger number of people. However, while previous reports suggest that the demand is increasing in relation to pets, our findings do not support this, and recent seizures suggest that the market is shifting towards or already exists for farmed animals. More research is needed specifically in relation to veterinary medicines and farmed animals. Regarding pets, to address the illicit market in veterinary medicines and avoid the risks associated with it, our research suggests practitioners and authorities increase their efforts to educate pet guardians about which sites they can trust and how to identify them. At source and transit points, our research highlights the need for better regulation of this market, improved detection and enforcement at borders, and increased public awareness of the existence of an illegal veterinary medicine market and the warning signs of suspect online sites.

## Footnotes

1 “Illegal veterinary medicines” is used as an umbrella term to encompass all types of illegally distributed veterinary pharmaceuticals.
